# Supplementary material for: Splice-Junction-Based Mapping of Alternative Isoforms in the Human Proteome
Source: Cell Rep. Author manuscript; Available in PMC 2020 Jan 15. (PMC6961840; doi:10.1016/j.celrep.2019.11.026)
Supplement: 2 [file NIHMS1546469-supplement-2.zip › DF1/PXD010154/Heart/Heart_3_TTN_KPEAPPPKVEETPEEIIYEEK.pdf]

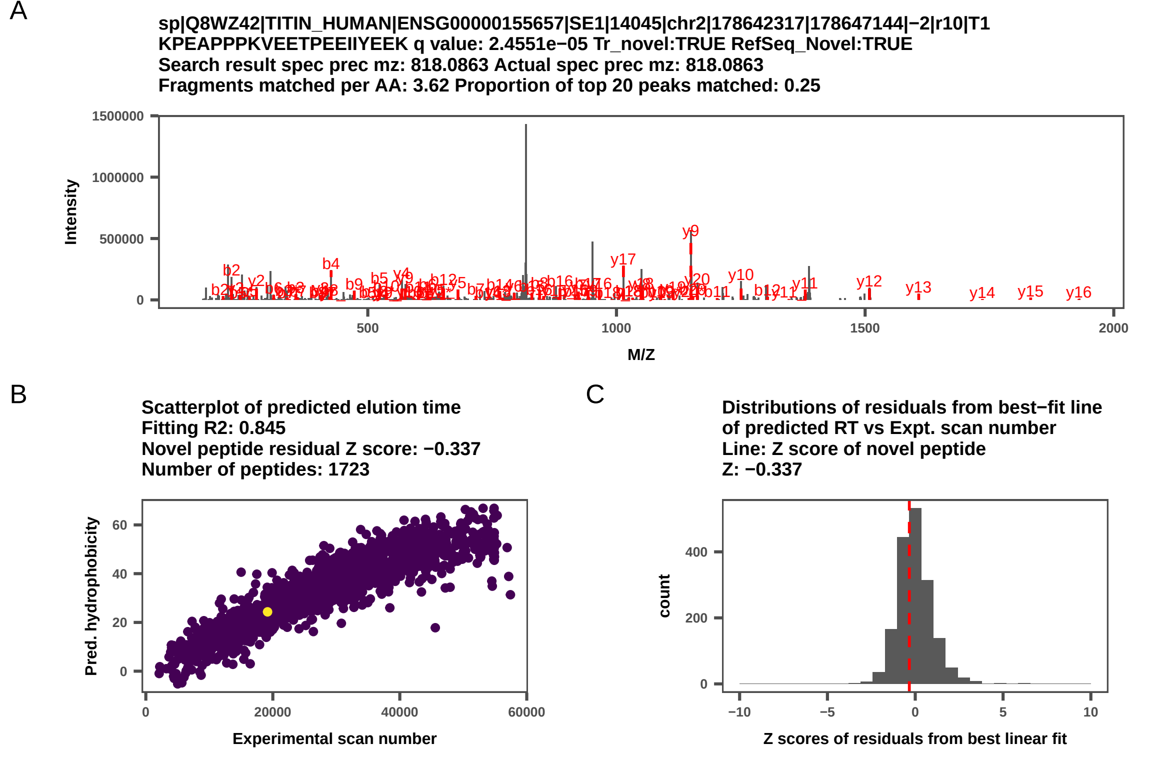
A
sp|Q8WZ42|TITIN_HUMAN|ENSG00000155657|SE1|14045|chr2|178642317|178647144|−2|r10|T1
KPEAPPPKVEETPEEIIYEEK q value: 2.4551e−05 Tr_novel:TRUE RefSeq_Novel:TRUE
Search result spec prec mz: 818.0863 Actual spec prec mz: 818.0863
Fragments matched per AA: 3.62 Proportion of top 20 peaks matched: 0.25
Intensity
1500000
1000000
500000
0
M/Z
500
1000
1500
2000
b2
b4
y2
b5
b9
y9
y17
y20
y10
y11
y12
y13
y14
y15
y16
B
Scatterplot of predicted elution time
Fitting R2: 0.845
Novel peptide residual Z score: −0.337
Number of peptides: 1723
Pred. hydrophobicity
60
40
20
0
Experimental scan number
0
20000
40000
60000
C
Distributions of residuals from best−fit line
of predicted RT vs Expt. scan number
Line: Z score of novel peptide
Z: −0.337
count
400
200
0
Z scores of residuals from best linear fit
−10
−5
0
5
10
